# Gene set proximity analysis: expanding gene set enrichment analysis through learned geometric embeddings, with drug-repurposing applications in COVID-19

**DOI:** 10.1093/bioinformatics/btac735

**Published:** 2022-11-17

**Authors:** Henry Cousins, Taryn Hall, Yinglong Guo, Luke Tso, Kathy T H Tzeng, Le Cong, Russ B Altman

**Affiliations:** Department of Biomedical Data Science, Stanford University School of Medicine, Stanford, CA 94305, USA; Optum Labs at UnitedHealth Group, Minneapolis, MN 55343, USA; Optum Labs at UnitedHealth Group, Minneapolis, MN 55343, USA; Optum Labs at UnitedHealth Group, Minneapolis, MN 55343, USA; Optum Labs at UnitedHealth Group, Minneapolis, MN 55343, USA; Department of Genetics, Stanford University School of Medicine, Stanford, CA 94305, USA; Department of Pathology, Stanford University School of Medicine, Stanford, CA 94305, USA; Department of Biomedical Data Science, Stanford University School of Medicine, Stanford, CA 94305, USA; Department of Genetics, Stanford University School of Medicine, Stanford, CA 94305, USA; Department of Medicine, Stanford University School of Medicine, Stanford, CA 94305, USA; Department of Bioengineering, Stanford University, Stanford, CA 94305, USA

## Abstract

**Motivation:**

Gene set analysis methods rely on knowledge-based representations of genetic interactions in the form of both gene set collections and protein–protein interaction (PPI) networks. However, explicit representations of genetic interactions often fail to capture complex interdependencies among genes, limiting the analytic power of such methods.

**Results:**

We propose an extension of gene set enrichment analysis to a latent embedding space reflecting PPI network topology, called gene set proximity analysis (GSPA). Compared with existing methods, GSPA provides improved ability to identify disease-associated pathways in disease-matched gene expression datasets, while improving reproducibility of enrichment statistics for similar gene sets. GSPA is statistically straightforward, reducing to a version of traditional gene set enrichment analysis through a single user-defined parameter. We apply our method to identify novel drug associations with SARS-CoV-2 viral entry. Finally, we validate our drug association predictions through retrospective clinical analysis of claims data from 8 million patients, supporting a role for gabapentin as a risk factor and metformin as a protective factor for severe COVID-19.

**Availability and implementation:**

GSPA is available for download as a command-line Python package at https://github.com/henrycousins/gspa.

**Supplementary information:**

Supplementary data are available at *Bioinformatics* online.

## 1 Introduction

High-throughput sequencing and genetic perturbation methods produce vast functional genetic datasets representing both clinical and molecular phenotypes, with applications from drug discovery to clinical diagnostics (Cieślik and Chinnaiyan, 2017). While gene-level expression data are useful for identifying differentially expressed genes or training predictive models, assigning biological relevance to observed expression patterns generally requires inference of perturbation in higher-order functional pathways (De Leeuw *et al.*, 2016). This requires both defining the composition of such pathways and recognizing subtle expression signatures in noisy data.

Methods for analyzing expression changes in predefined gene sets generally fall into one of two algorithmic classes. These include overrepresentation approaches, which detect pathway enrichment within a set of differentially expressed genes, and aggregate-score approaches, which associate input genes with a continuous phenotypic score. Overrepresentation approaches, such as Database for Annotation, Visualization, and Integrated Discovery (DAVID) and Enrichr, benefit from simpler input requirements and faster runtimes due to compatibility with deterministic significance tests (Boyle *et al.*, 2004; Chen *et al.*, 2013; Huang *et al.*, 2009). However, the requirement for a binary threshold for differential expression limits their ability to detect pathway changes resulting from weaker differential expression among many genes, as well as the relative ordering of genes.

Aggregate-score approaches, in contrast, weight gene sets according to a cumulative statistic based on continuous phenotypic scores for genes. The most popular aggregate-score approach is gene set enrichment analysis (GSEA), which computes a weighted Kolmogorov–Smirnov statistic representing enrichment of a gene set in an ordered gene list (Subramanian *et al.*, 2005). This enrichment statistic is then compared with a null distribution generated from random permutations of the genes or phenotypes to assign a significance level. Aggregate-score approaches such as GSEA, Correlation Adjusted Mean Rank (CAMERA) and Pathway Analysis with Down-weighting of Overlapping Genes (PADOG) are more sensitive to large ensembles of genes acting in concert but only consider overlap between explicitly defined genes in a set and input list (Tarca *et al.*, 2012; Wu and Smyth, 2012). As a result, such methods cannot detect pathway perturbations implied from expression changes in neighboring genes, which is particularly important in the case of noisy datasets or incomplete gene sets (Goeman and Bühlmann, 2007).

To overcome these limitations, some gene set analysis methods incorporate a priori assumptions about genetic relationships, typically in the form of protein–protein interaction (PPI) networks (Hillenmeyer *et al.*, 2016; Szklarczyk *et al.*, 2017). This enables gene-level enrichment statistics to be augmented by expression measures of a local genetic neighborhood and can also improve visualization of enrichment results (Merico *et al.*, 2010). Due to the computational expense of repeatedly traversing large graphs such as PPI networks, nearly all network-based methods use the overrepresentation approach (Miryala *et al.*, 2018). Furthermore, such methods generally utilize explicit representations of network topology, such as one-hop neighbors of differentially expressed genes, which are limited in their ability to capture latent local and global network features. As a result, gene set analysis methods remain sensitive both to experimental noise and to the specific composition of gene sets under consideration, even among gene sets denoting the same pathway.

We hypothesize that capturing the full extent of functional pathway enrichment in an expression dataset requires consideration of complex pathway structures that are not efficiently represented in explicit form. Unsupervised representation learning provides a means of capturing such features objectively and has enabled improved definition and prognostic application of gene sets (Chen *et al.*, 2018; Wang *et al.*, 2020). However, incorporating latent embeddings into enrichment testing algorithms remains an open question, as the inter-gene dependencies implicit in such embeddings can confound relative significance estimates for the resulting gene sets under traditional methods (Goeman and Bühlmann, 2007; Wang *et al.*, 2020). Therefore, we propose a generalization of the classical GSEA algorithm to a latent feature space derived from unsupervised embeddings of a PPI network. Our method, called gene set proximity analysis (GSPA), implicitly considers the full network context of individual genes, allowing detection of implied perturbations in bona fide functional pathways. Notably, GSPA is statistically straightforward, reducing precisely to the GSEA algorithm for ranked gene lists through a single parameter. We find that GSPA outperforms both GSEA and two widely used network-augmented gene set analysis methods in identifying disease-associated pathways from gold-standard expression datasets, while also improving reproducibility for semantically similar gene sets.

Finally, we apply GSPA to a collection of datasets measuring gene involvement in SARS-CoV-2 viral entry using gene sets representing known targets of Food and Drug Administration (FDA)-approved drugs, identifying four common drugs (gabapentin, metformin, lorazepam and clonazepam) as possible modulators of SARS-CoV-2 viral entry. We subsequently investigate our predictions through propensity-score matched, retrospective analysis of health insurance claims from 8 million patients. Consistent with the results from GSPA, our clinical investigation supports a role for gabapentin and metformin as risk and protective factors for severe SARS-CoV-2 infection, respectively, providing insight into both molecular pathogenesis and potential treatment strategies for COVID-19.

## 2 Materials and methods

### 2.1 Generating gene embeddings

To generate representative embeddings for protein-coding genes, we obtained high-confidence PPIs from the STRING repository (version 11; Szklarczyk *et al.*, 2017). We included only interactions with a confidence score of at least 900 in humans, resulting in a network of 12 396 proteins and 324 152 interactions. This was represented as an undirected graph, *G* = (*V*, *E*), with *V*, the set of vertices, representing unique proteins and *E*, the set of edges, representing pairwise PPIs. Low-dimensional feature learning was performed on *G* using the node2vec algorithm, which learns node embeddings by simulating biased random walks in *G* to preserve local neighborhood architecture (Grover and Leskovec, 2016).

We assessed the functional relevance of the embeddings first by retrieving gene ontology (GO) cellular component (CC) labels for each gene (release 2022-07-01), identifying all genes uniquely defined by location in one of cytosol (GO: 0005829), plasma membrane (GO: 0005886), extracellular region (GO: 0005576) or nucleus (GO: 0005634) (Ashburner *et al.*, 2000; Carbon *et al.*, 2021). To visualize embeddings qualitatively, we performed *t*-distributed stochastic neighbor embedding (tSNE) with perplexity 80, overlaying embeddings with their corresponding GO-CC terms. Next, we retrieved GO biological process (BP) terms for all genes and defined inter-gene similarity as the Jaccard index of the GO-BP term sets for a given pair of genes. Using a random sample of 100 000 gene pairs and their corresponding GO-BP similarity, we labeled gene pairs as sharing low (≤5th percentile), medium (25th–75th percentile) or high (≥95th percentile) functional similarity and compared the distribution of cosine distances between their embeddings. Similarly, we compared the distribution of cosine distances between embeddings for gene pairs based on the length of their shortest path (1, 2 or ≥3). In both cases, Mann–Whitney *U* tests were used for comparing distributions. While we developed and tested GSPA using the embeddings described above, we also note that the algorithm is interoperable with gene embeddings derived by other methods, enabling users to fine-tune embeddings for their desired tasks.

### 2.2 Calculation of enrichment score

**Fig. 1.**
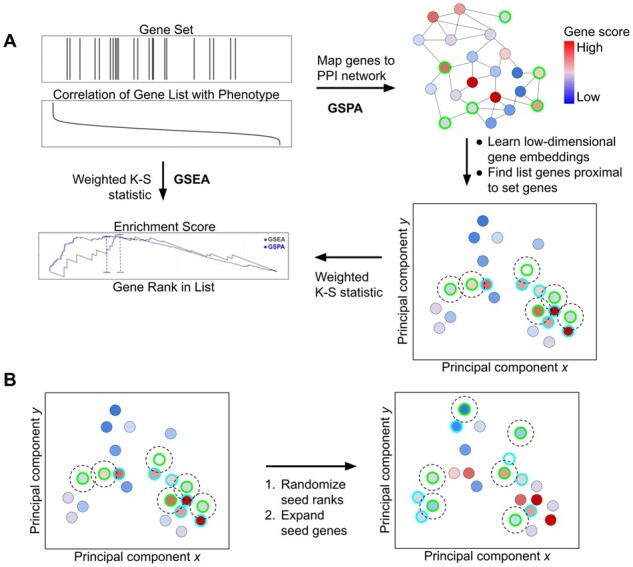
Overview of GSPA algorithm. (**A**) Beginning with a gene set and a list of genes ranked by a continuous score, GSPA maps gene IDs to precomputed embeddings representing the genes’ positions within a human PPI network. A set of genes (outlined in cyan) proximal to the original gene set (outlined in green) is determined by adding genes that are within a specified distance, in latent space, from any gene in the original set. An enrichment statistic is computed as the weighted K–S statistic of the augmented gene set. (**B**) For every gene set, a null distribution is determined by performing a fixed number of random permutations of the original gene set, then performing the latent expansion step to incorporate proximal genes

For a given gene set and ranked gene list, the enrichment score (ES) in GSPA is computed in a similar manner as in GSEA, with an important modification (Fig. 1A). In GSEA, a weighted running sum statistic is computed by walking down the ranked list, incrementing the statistic in proportion to its phenotypic score when encountering a gene in the gene set and decrementing it otherwise. The ES is then the signed maximum value of the statistic. Equivalently,

(1) $$\begin{array}{c} ES_{k}^{\mathrm{GSEA}}=\underset{1\leq i\leq n}{\mathrm{maxdev}}\left(\frac{\sum_{t=1}^{i}s_{t}\cdot 1_{g_{t}\in G_{k}}}{\sum_{t=1}^{n}s_{t}\cdot 1_{g_{t}\in G_{k}}}-\frac{\sum_{t=1}^{i}1_{g_{t}\notin G_{k}}}{n-\left|G_{k}\right|}\right), \end{array}$$

where ${\text{ES}}_{k}^{\text{GSEA}}$ is the ES for gene set *G_k_*, maxdev is the maximum deviation from zero, *|G_k_|* is the number of list genes in the gene set, *i* represents a position in the ranked gene list, *n* is the number of genes in the gene list, *s_t_* is the value of the phenotypic score for a given gene *g_t_* and 1 is the indicator function for membership of *g_t_* with respect to *G_k_*.

In GSPA, while calculating the running sum statistic, the gene set *G_k_* is temporarily augmented to create the set of gene-set-proximal genes *P_k_* such that

(2) $$\begin{array}{c} P_{k}=\{g\in L:\min\left(\mathrm{dist}\left(v_{g},V_{G_{k}}\right)\right)\leq r\}, \end{array}$$

where *g* represents any gene in the ranked list *L*, *v_g_* represents the embedding for *g*, *V_Gk_* represents a list of embeddings for each gene in *G_k_*, dist outputs a list of cosine distances from *v_g_* to each embedding in *V_Gk_*, assuming that unique embeddings are available for all genes, and *r* is a user-defined parameter representing the radius by which to expand each member of the original gene set. The default radius is 0.1, corresponding to the distance at which approximately 50% of 1-hop neighbors are included. The raw ES is then computed as in Equation (1), substituting *P_k_* for *G_k_*.

(3) $$\begin{array}{c} ES_{k}^{\mathrm{GSPA}}=\underset{1\leq i\leq n}{\mathrm{maxdev}}\left(\frac{\sum_{t=1}^{i}s_{t}\cdot 1_{g_{t}\in P_{k}}}{\sum_{t=1}^{n}s_{t}\cdot 1_{g_{t}\in P_{k}}}-\frac{\sum_{t=1}^{i}1_{g_{t}\notin P_{k}}}{n-\left|P_{k}\right|}\right). \end{array}$$

Of note, this reduces to Equation (1), the definition of ES in GSEA, as *r* decreases to zero.

### 2.3 Generating null distributions

The generation of a null distribution of ESs for a given gene set is important both for assigning relative rankings to gene sets of different sizes and for assigning significance levels. In the GSEA algorithm for pre-ranked gene lists, null distributions are generated by sampling a random gene set *G_k_′* containing the same number of members in the ranked list as the original set *G_k_* and recalculating ES. This implicitly defines a null hypothesis of no association between genes, which, for large gene sets, can result in highly sensitive estimates of significance at the expense of specificity. Therefore, by default, GSPA generates null distributions by first resampling the original gene set to create *G_k_′*, then creating a null set of proximal genes *P_k_′* as in the original ES calculation for GSPA (Fig. 1B). A null ES is defined from *P_k_′,* and this procedure is repeated a fixed number of times (100 by default). Alternatively, users can test a less stringent null hypothesis by directly resampling *P_k_* itself. Both methods constitute hybrid null hypotheses (i.e. that relative gene expression patterns do not differ between the gene set and background genes) that reduce precisely to the original GSEA prerank algorithm as *r* decreases to zero, but the former method directly accounts for known correlations between genes (Maleki *et al.*, 2020). Once the ES and null ES distribution have been calculated, normalized ES (NES; a normalized transformation of ES accounting for gene set size), *P*-value and false discovery rate (FDR) are calculated as in GSEA (Subramanian *et al.*, 2005).

### 2.4 Datasets

To evaluate the performance of GSPA, we used the gold-standard GEO2KEGG compendium, composed of 42 disease-matched, human gene expression profiles obtained from the Bioconductor package (version 3.13; Supplementary Table S1; Tarca *et al.*, 2012, 2013). Each dataset contains microarray results from an Affymetrix platform and is mapped to 1 of 19 different diseases. Furthermore, each disease is accompanied by a predefined set of disease-associated Kyoto Encyclopedia of Genes and Genomes (KEGG) pathways obtained from the MalaCards database of disease–gene associations (Geistlinger *et al.*, 2021). These datasets have been used in previous comparisons of gene set analysis methods (Geistlinger *et al.*, 2021, 2016; Tarca *et al.*, 2013).

For running GSPA, KEGG human pathway gene sets were obtained through the Bioconductor EnrichmentBrowser package (version 2.22.2; Geistlinger *et al.*, 2016; Kanehisa *et al.*, 2017). Gene sets containing fewer than 2 or greater than 300 protein-coding genes were excluded from all analyses, resulting in a total of 333 KEGG gene sets considered.

### 2.5 Running alternative enrichment testing methods

We compared our approach to three alternative enrichment analysis methods. In addition to GSEA itself, we also tested network-based GSEA (NGSEA), a network-augmented method accepting continuous gene scores as inputs, and EnrichNet, a network-based method accepting gene sets as inputs (Glaab *et al.*, 2012; Han *et al.*, 2019). NGSEA operates by considering aggregate expression changes in 1-hop PPI network neighborhoods, rather than individual genes, while EnrichNet calculates a network distance metric between specific sets of genes. We ran the GSEA prerank method with the default parameters using the Java implementation (version 3.0). We ran NGSEA with the default parameters using the NGSEA web server (https://www.inetbio.org/ngsea/index.php; accessed June 2021). We ran EnrichNet with the default parameters using the EnrichNet web server (http://www.enrichnet.org/, accessed July 2022), defining the top 5% of genes as differentially expressed.

### 2.6 Benchmarking prediction of disease-associated gene sets

To measure the ability of GSPA to identify disease-associated pathways, we used a version of the procedure proposed by Geistlinger *et al.* (2021) for evaluating the biological relevance of gene set analysis methods. For each disease represented in our collection of evaluation datasets, we established a set of ‘ground-truth’ KEGG pathways known to exhibit a strong association with the disease (specifically, a positive relevance score in the GSEABenchmarkeR package). We subsequently ran GSPA, GSEA, NGSEA and EnrichNet on each expression dataset in the GEO2KEGG compendium using KEGG pathway gene sets. For each method, KEGG pathways were percentile-ranked by the method’s primary enrichment statistic (NES for GSPA, GSEA and NGSEA and XD-score for EnrichNet), with higher rankings connoting greater significance (Geistlinger *et al.*, 2016). We then measured the ability of each method’s ranking to retrieve ‘ground-truth’ gene sets using area under the precision–recall curve (AUPRC). Significance levels for each method were calculated using Wilcoxon signed-rank tests.

### 2.7 Benchmarking reproducibility of gene set rankings

To measure the extent to which similar rankings are preserved for gene sets representing the same pathway, we first defined a set of 16 unique, semantically similar KEGG gene set pairs. Specifically, we defined semantically similar gene sets as unique pairs where at least one of the descriptions, as word sets, completely included the other. For instance, hsa00100 (‘steroid_biosynthesis’) was matched with hsa00140 (‘steroid_hormone_biosynthesis’) and hsa04136 (‘autophagy’) was matched with hsa04140 (‘autophagy’). The complete list of gene set pairs is included in Supplementary Table S2. For each of the GEO2KEGG datasets, we then computed gene set rankings based on NES for GSPA, GSEA, NGSEA and EnrichNet and compared the similarity between predictions for matched gene sets by Spearman rank correlation coefficient, comparing methods’ performance by Wilcoxon signed-rank tests.

### 2.8 Prioritizing pharmacologic modulators of SARS-CoV-2 entry

To identify FDA-approved compounds with potential relevance in COVID-19 drug repurposing efforts, we obtained drug-target gene sets from the DSigDB D1 database (version 1.0), representing sets of known targets for FDA-approved drugs (Yoo *et al.*, 2015). We obtained ranked gene lists from three genome-wide CRISPR knock-out screens of host gene importance for SARS-CoV-2 viral entry, for which additional details regarding experimental design and data processing are available at the respective citations (Daniloski *et al.*, 2021; Schneider *et al.*, 2021; Wei *et al.*, 2021). We performed GSPA separately on each dataset using DSigDB D1 gene sets and default GSPA parameters. For subsequent clinical validation, we restricted analysis to commonly prescribed drugs, defined as within the top 100 most commonly prescribed medications in 2020 in the USA (ClinCalc DrugStats Database, https://clincalc.com/DrugStats, version 21.1).

### 2.9 Claims database and cohort construction

The study sample for clinical analysis was obtained from de-identified administrative claims for Medicare Advantage Part D (MAPD) enrollees in a research database (Kelly *et al.*, 2021; Wallace *et al.*, 2014; Yao *et al.*, 2016). The database contains medical (emergency, inpatient and outpatient diagnoses and treatments) and pharmacy claims for services submitted for third-party reimbursement, available as International Classification of Diseases, Tenth Revision, Clinical Modification (ICD-10-CM) and National Drug Codes claims, respectively. These claims were aggregated after completion of care encounters and submission of claims for reimbursement.

For each drug of interest, we constructed a cohort of individuals with at least 11 months of enrollment in MAPD insurance from January to December 2019 and at least 1 month of enrollment in MAPD in 2020. These individuals had at least one pharmacy prescription claim during their enrollment and lived in counties in New York, New Jersey and Connecticut. In our database, COVID-19 hospitalization was more prevalent among individuals insured through MAPD and among residents of the New York, New Jersey and Connecticut tri-state area. We restricted our analyses to these populations to select for uniform exposure to COVID-19 and a higher prevalence of the COVID-19 hospitalization outcome in our cohort. We defined our outcome as a claim for a hospitalization with a positive COVID-19 test between January 1, 2020 and June 26, 2020 (Supplementary Table S3).

Prescription drug users were identified by string matching from pharmacy claims for any of the generic names associated with the drug candidate. We considered individuals to be drug-exposed when their total supply days covered ≥80% of days between their first drug use date after July 1, 2019 to January 31, 2020. We considered individuals non-drug-exposed if the individual was never prescribed the drug candidates or drugs in the same therapeutic class, between July 1, 2019 and January 31, 2020. For each drug of interest, we extracted covariates for both drug-exposed individuals and non-drug-exposed individuals, a full description of which is available in Supplementary Table S4.

### 2.10 Controlled study without propensity score matching

We first selected a list of features using a least absolute shrinkage and selection operator (LASSO) model with tuned penalty coefficient based on Bayesian information criteria. The complete list of features includes normalized age, sex, primary treatment-related diagnosis, comorbidity index flags, occurrence flags to first three digits of diagnosis codes, adherence flags to co-used drug therapeutic classes, race, state of residence and normalized socioeconomic status (SES) index. After feature selection, we added normalized age, normalized SES index and primary treatment-related diagnosis into the feature list to control for these factors. To ensure model convergence, we excluded features with a prevalence of less than 1% of the cohort. We then fit a Cox proportional hazard model to determine the adjusted hazard ratio of the treatment group, considering time to COVID-19 hospitalization, controlling for the list of features selected.

### 2.11 Controlled study with propensity score matching

For the group of drug-exposed individuals, we applied 1:1 propensity score matching (PSM) to construct a matched group of non-drug-exposed individuals. The propensity score was built using logistic regression based on age, sex, primary treatment-related diagnosis, comorbidity index flags, occurrence flags to first three digits of diagnosis codes, adherence flags to co-used drug therapeutic classes, race, state of residence and SES index. We ran 1:1 PSM with a caliper of 0.25 multiplied by the standard deviation (SD) of propensity scores. We assessed PSM performance by calculating the standardized mean difference between drug-exposed and non-exposed groups across the primary treatment-related diagnosis. PSM was considered adequate when the standardized mean difference between groups was ≤0.10 (Zhang *et al.*, 2019). We applied the same procedure of feature selection and similarly fit a Cox proportional hazards model for each drug of interest, between baseline (the state-specific time of first COVID-19 hospitalization) to hospitalization or end of follow-up, to investigate the adjusted hazard ratio of the drug-exposed group. We applied a Benjamini–Hochberg correction with FDR 0.1 to control for multiple hypothesis testing.

### 2.12 Code and data availability

A complete implementation of GSPA, including precomputed embeddings, is available as a command-line Python program at https://github.com/henrycousins/gspa.

## 3 Results

### 3.1 Overview of GSPA

Network topology-based gene set analysis methods rely on the principle that aggregate expression changes in gene sets are better resolved by considering expression changes in local gene subnetworks (Fig. 1). GSPA extends this principle to a learned latent space that reflects genes’ functional similarity through a low-dimensional representation of their contexts in a complete PPI network. GSPA calculates the enrichment of a gene set in a ranked list in an analogous manner to GSEA, leveraging the principle that genes with highly similar embeddings are likely to share functional overlap. Specifically, GSPA computes an ES through the same weighted Kolmogorov–Smirnov statistic used in GSEA, but considering the union of the original gene set and the set of proximal genes meeting a predefined level of embedding similarity to any member of the original gene set.

**Fig. 2.**
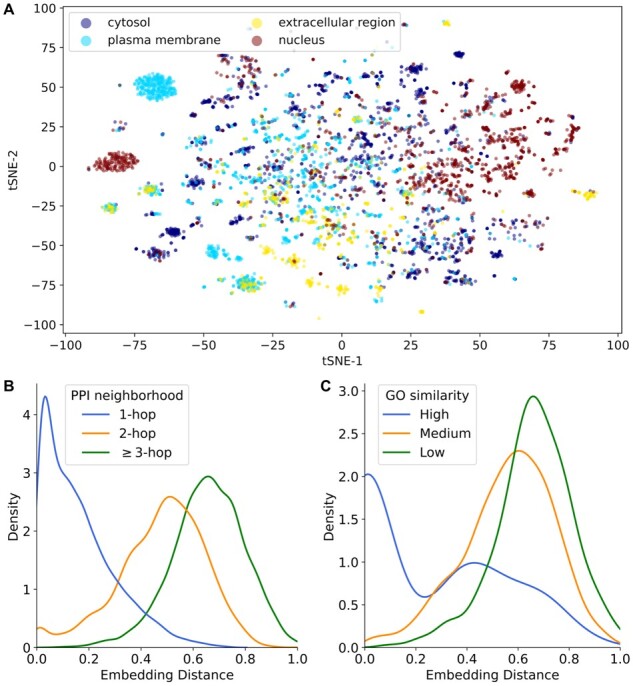
Visualization of unsupervised gene embeddings. (**A**) tSNE projections of gene embeddings, colored by GO-CC term association. (**B**) Distribution of embedding distances for pairs of n-hop neighbors in the PPI network. Gene pairs with lower shortest paths have more similar embeddings. (**C**) Distribution of embedding distances for pairs of genes sharing high (≥95th percentile), medium (25th–75th percentile) or low (≤5th percentile) similarity of GO-BP terms, measured by Jaccard similarity. Genes with greater GO-BP similarity have more similar embeddings

To confirm that the PPI embeddings (Fig. 2A) captured biologically meaningful signals, we measured the relationship between inter-gene embedding distances and both PPI network neighborhood (Fig. 2B) and GO-BP functional similarity (Fig. 2C), finding that lower cosine distances between embeddings were associated with both lower shortest paths in the PPI network and higher similarity of GO-BP terms (*P* < 0.001 for all comparisons).

### 3.2 GSPA outperforms existing methods in predicting disease-associated gene sets from expression data

**Fig. 3.**
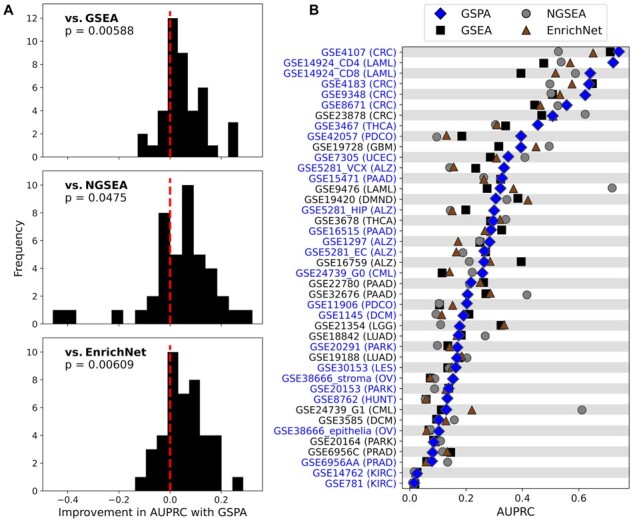
Ability to retrieve known disease-associated gene sets. (**A**) Differences in AUPRC on each dataset between GSPA and GSEA, NGSEA and EnrichNet for identification of literature-derived, disease-associated gene sets in disease-specific expression datasets. Histogram density to the right of the red line indicates better performance from GSPA. (**B**) Performance of GSPA (blue), GSEA (black), NGSEA (gray) and EnrichNet (brown) on all individual datasets. Blue text indicates where GSPA was among the top two best-performing methods

We evaluated the ability of GSPA, in addition to GSEA, NGSEA and EnrichNet, to identify known disease-associated gene sets from gene expression datasets for the corresponding diseases (Fig. 3). For this analysis, we used the gold-standard GEO2KEGG compendium, containing 42 gene expression datasets matched to 19 different diseases. Each disease was matched with a set of known disease-associated KEGG pathways previously defined by Geistlinger *et al.* (2021) for use in evaluating gene set analysis methods. For each expression dataset, we ranked human KEGG pathways using GSPA, GSEA, NGSEA and EnrichNet and measured each method’s ability to identify known disease-associated pathways.

We observed significantly higher predictive ability with GSPA compared with GSEA, NGSEA and EnrichNet (*P* = 0.00588, *P* = 0.0475 and *P* = 0.00609, respectively; Fig. 3A). The mean AUPRC was 0.285 (SD 0.186) using GSPA, 0.250 (SD 0.161) using GSEA, 0.269 (SD 0.191) using NGSEA and 0.250 (SD 0.167) using EnrichNet. GSPA outperformed GSEA on 27 of the 42 tested datasets (64.3%), NGSEA on 28 datasets (66.7%) and EnrichNet on 28 datasets (66.7%; Fig. 3B). It scored highly on datasets for a variety of diseases, including both solid and liquid cancers and chronic pulmonary disease, although performance was relatively reduced in Parkinson’s and cardiomyopathy datasets.

### 3.3 GSPA outperforms existing methods in reproducing rankings for semantically similar gene sets

**Fig. 4.**
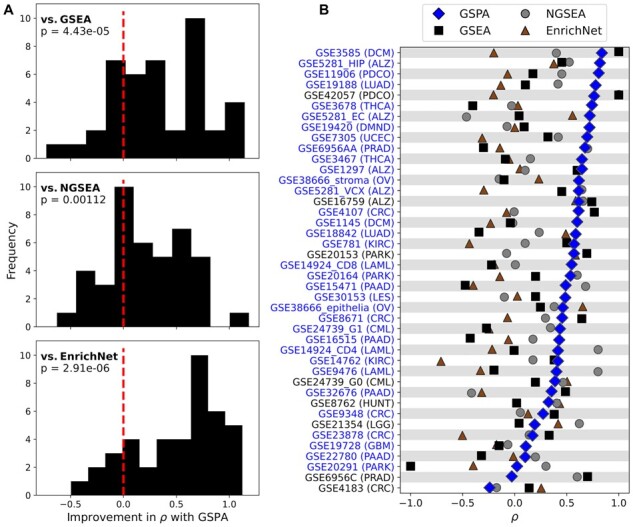
Reproducibility among semantically similar gene sets. (**A**) Differences in correlation on each dataset among GSPA and GSEA, NGSEA and EnrichNet between rankings of matched gene sets representing the same pathway. Histogram density to the right of the red line indicates better performance from GSPA. (**B**) Performance of GSPA (blue), GSEA (black), NGSEA (gray) and EnrichNet (brown) on all individual datasets. Blue text indicates where GSPA performed either best or second-best

We next assessed the ability of GSPA to return semantically consistent results (Fig. 4), as traditional gene set analysis methods are sensitive to the specific member-wise composition of a gene set, which is often arbitrary with respect to the gene set’s intended meaning. We used an automated procedure to define semantically similar gene-set pairs, such as ‘hsa00100_Steroid_biosynthesis’ and ‘hsa00140_Steroid_hormone_ biosynthesis’, and measured the pairwise correlation between enrichment rankings for each method (Supplementary Table S2). We observed significantly stronger correlations with GSPA than with GSEA, NGSEA or EnrichNet (*P* = 4.43*e*−5, *P* = 0.00112 and *P* = 2.91*e*−6, respectively; Fig. 4A). The mean correlation was 0.485 (SD 0.245) using GSPA, 0.156 (SD 0.425) using GSEA, 0.268 (SD 0.333) using NGSEA and −0.0153 (SD 0.335) using EnrichNet. GSPA outperformed GSEA on 31 of the 42 tested datasets (73.8%), NGSEA on 27 datasets (64.3%) and EnrichNet on 35 datasets (83.3%; Fig. 4B).

### 3.4 GSPA enables prediction of pharmacologic modulators of SARS-CoV-2 viral entry

**Fig. 5.**
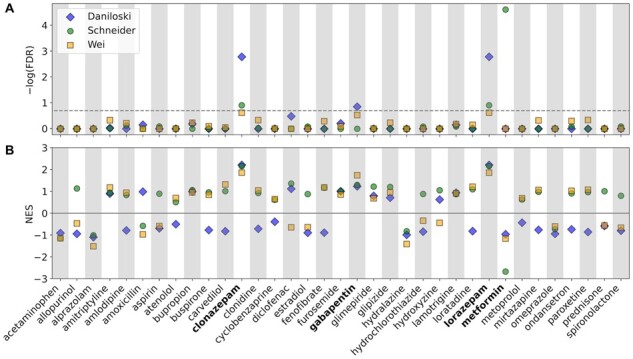
Analysis of SARS-CoV-2 entry datasets using DSigDB gene sets. (**A**) GSPA FDR scores for commonly prescribed, FDA-approved drugs on three datasets of gene essentiality for SARS-CoV-2 viral entry. Dashed line indicates an FDR threshold of 0.5. (**B**) Same as (A) but showing NES. Positive and negative NES indicate opposite predicted effects

Gene set analysis methods are valuable tools for drug discovery and repurposing, as they provide a means of associating specific pharmacologic agents with disease phenotypes by means of aggregate expression changes in gene ensembles targeted by the same compound. Therefore, as a demonstration of our model’s utility, we next investigated whether GSPA could identify novel drug repurposing opportunities in COVID-19. Given the growing literature supporting a multifactorial viral entry mechanism influenced by many host genes, we focused on identifying approved drugs able to modulate viral entry into host cells (Daniloski *et al.*, 2021; Schneider *et al.*, 2021; Wei *et al.*, 2021). We obtained three datasets representing genome-wide CRISPR knock-out screens ranking host genes by their associated effect on SARS-CoV-2 viral entry. We ran GSPA separately on each dataset with drug-target gene sets from the DSigDB D1 library, generating rankings of FDA-approved drugs by screen-specific enrichment. Notably, the three datasets revealed an enrichment of drug classes with documented COVID-19 associations, including anticonvulsants and biguanides (Li *et al.*, 2021; Luo *et al.*, 2020; Zhang *et al.*, 2021). For follow-up analysis, we restricted our search to highly prescribed drugs (within the top 100 in 2020 in the USA), of which only four drugs had a FDR below 0.5 in any dataset: the benzodiazepines clonazepam and lorazepam, gabapentin and metformin (Fig. 5A). Furthermore, we noted that gabapentin, clonazepam and lorazepam were associated with positive NES values, while NES values for metformin were consistently negative (Fig. 5B). This implies that metformin could exert an opposite effect on viral entry in comparison to gabapentin, clonazepam and lorazepam.

### 3.5 Retrospective analysis of health insurance claims supports a role for metformin and gabapentin in modulating SARS-CoV-2 viral entry

**Fig. 6.**
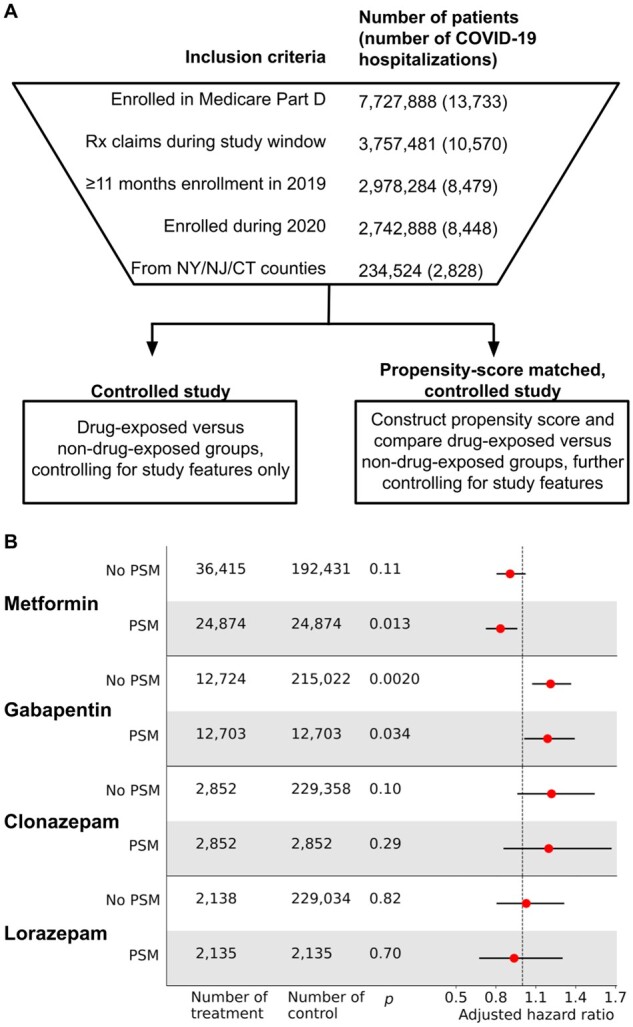
Retrospective clinical analysis of drug effect on COVID-19 hospitalization. (**A**) Overview of analysis procedure. From 7.7 million database patients, 234 524 met criteria for inclusion. Two comparisons were performed, comparing COVID-19 hospitalization rates among drug-exposed versus non-drug-exposed patients, controlling for covariates, with and without PSM. (**B**) Results for drug hits identified by GSPA. Metformin shows a significant negative association with hospitalization, while gabapentin shows a significant positive association with hospitalization

To investigate whether prediction of a modulatory effect on SARS-CoV-2 viral entry correlates with a drug’s clinical effect, we performed a retrospective analysis of claims data from a large US health insurance provider, examining associations between common prescriptions and COVID-19 hospitalization rates (Fig. 6A). We reviewed claims from 7.7 million MAPD members for compatibility with regional and temporal inclusion criteria. The final dataset comprised claims for 234 524 MAPD-insured residents of New York, New Jersey and Connecticut with at least 11 months of enrollment between January and December 2019 and at least 1 month of enrollment during 2020, with at least one pharmacy prescription claim. Among these individuals, 2828 (1.21%) had claims indicating COVID-19 hospitalization during the observation window.

For each of the four candidate drugs, we measured the association between drug use and hospitalization due to COVID-19 both with and without 1:1 PSM (Fig. 6B). After controlling for multiple hypothesis testing, neither clonazepam nor lorazepam was associated with a significant hazard ratio for COVID-19 hospitalization. However, gabapentin use was associated with a significantly elevated risk of COVID-19 hospitalization, with an adjusted hazard ratio of 1.211 (*P* = 0.0020) before and 1.189 (*P* = 0.034) after applying PSM. Furthermore, after applying PSM, metformin use was associated with a reduction in COVID-19 hospitalization risk, with an adjusted hazard ratio of 0.834 (*P* = 0.013).

## 4 Discussion

In this work, we present a novel algorithm, GSPA, based on unsupervised graph learning that extends traditional gene set enrichment analysis to a latent feature space reflecting complex interactions among protein-coding genes. While some existing methods for gene set analysis use a priori knowledge of pathway structures in the form of PPI networks, most such methods work by measuring distance between two distinct sets of genes, requiring thresholding of experimental measurements to establish a set of differentially expressed genes, as in the case of EnrichNet (Glaab *et al.*, 2012; Tarca *et al.*, 2009). Of the few network-augmented methods that consider continuous experimental scores, all use explicit representations of network structure, requiring either consideration of only the immediate neighborhood of a gene of interest, as in the case of NGSEA (Han *et al.*, 2019), or long runtimes (Nadeau *et al.*, 2021). GSPA derives from the theory that unsupervised graph embeddings, popular in social network analysis, provide lightweight and expressive representations of PPI network topology. This notion provides a demonstrated basis for identifying disease biomarkers and functional gene set members (Maddouri *et al.*, 2022; Wang *et al.*, 2020). However, simply applying GSEA methods to the enhanced gene sets resulting from network-based expansion methods results in non-specific significance estimates, as randomly derived null distributions fail to account for presumptive associations between expression changes for PPI network neighbors (Goeman and Bühlmann, 2007). The null distribution calculated by GSPA implicitly accounts for known associations, improving the biological relevance of the resulting pathway rankings.

To our knowledge, GSPA is the first aggregate-score gene set analysis method to incorporate implicit representations of pathway features, which provides several advantages in comparison to other network-augmented gene set analysis methods. First, learned embeddings capture both local and global genetic features that would not be apparent using simpler gene-level metrics such as shortest path length to a gene set. They are also modular, allowing any user-defined vector representation of gene similarity to be substituted, and require significantly less computational expense than do operations on unreduced networks. Furthermore, the use of embedding similarity to determine proximal genes enables a more conservative and biologically reasonable null hypothesis that accounts for known gene–gene associations, which is not possible in most gene set analysis methods for ranked lists. Finally, the GSPA algorithm accommodates arbitrary user-defined gene similarity thresholds, reducing to the traditional GSEA prerank algorithm as this parameter becomes highly stringent.

We apply GSPA to a variety of gene set analysis tasks, showing that it provides improved performance with respect to detection of disease-associated pathways in gene expression datasets, compared with GSEA and two widely used network-augmented approaches. GSPA achieved high performance across a variety of disease types, including solid cancers, leukemias and chronic pulmonary diseases. We observe that GSPA tends to perform worse on neurodegenerative diseases, which was also observed for NGSEA. We hypothesize that this phenomenon may derive from relative underrepresentation of these conditions in literature-based PPI networks, in comparison to cancers (Szklarczyk *et al.*, 2017). For specialized applications in specific diseases, fine-tuning gene embeddings based on cell-type-specific expression or disease-specific literature may improve performance in the future (Ietswaart *et al.*, 2021; Zhao *et al.*, 2021).

We also demonstrate that GSPA improves reproducibility of enrichment statistics for gene sets with a shared semantic meaning. A common problem in traditional gene set analysis methods is their sensitivity to small changes in the definition of knowledge-based gene sets. For instance, for the diabetes mellitus dataset assessed in the original GSEA report, the MSigDB C2 gene set ‘p38mapkPathway’ was ranked 20th in enrichment, while ‘ST_p38_MAPK_Pathway’ was ranked 117th out of 318 (Mootha *et al.*, 2003). Such variance reflects discrepancies between the specific definition of a gene set and the underlying pathway that it represents semantically, impairing both interpretability and reproducibility of enrichment analyses. By representing gene sets implicitly in a latent feature space, GSPA reduces the sensitivity of the enrichment test to these discrepancies.

Finally, we use GSPA to make novel predictions of drug associations with SARS-CoV-2 infection. We apply the algorithm on three datasets measuring genome-wide gene essentiality for viral entry in lung and epithelial host cells using gene sets representing known targets of FDA-approved drugs. This yielded four commonly prescribed drugs with a predicted modulatory effect on SARS-CoV-2 entry: metformin, gabapentin, clonazepam and lorazepam. While gabapentin, clonazepam and lorazepam targets were significantly enriched toward the top of the datasets, metformin targets were significantly enriched toward the bottom, suggesting an opposing effect of metformin in comparison to the other drugs.

We validated the drug predictions through retrospective analysis of COVID-19 hospitalization rates in propensity-score-matched subjects with or without exposure to each drug, observing statistically significant hazard ratios associated with both gabapentin and metformin. Specifically, we observed that gabapentin was associated with increased risk of COVID-19 hospitalization, while metformin was associated with decreased risk, consistent with the opposing effects predicted by GSPA. Several retrospective studies have demonstrated an association between metformin use and COVID-19 hospitalization and mortality, supporting the generalizability of our clinical findings (Crouse *et al.*, 2021; Lalau *et al.*, 2021; Luo *et al.*, 2020). However, the potential role of gabapentin as a risk factor for viral entry has not been investigated outside of our study. Further experimental and clinical investigations are necessary to clarify the potential effect size and mechanism of action of gabapentin in COVID-19 patients.

## Supplementary Material

btac735_Supplementary_Data

## Data Availability

The claims data are proprietary but, under certain conditions, may be made available through Optum Labs under a data use agreement. All other data are publicly available at their respective citations.
