# Supplementary material for: Gene set proximity analysis: expanding gene set enrichment analysis through learned geometric embeddings, with drug-repurposing applications in COVID-19
Source: Bioinformatics. 2022 Nov 17;39(1):btac735. doi: 10.1093/bioinformatics/btac735 (PMC9805577; doi:10.1093/bioinformatics/btac735)
Supplement: btac735_Supplementary_Data [file btac735_supplementary_data.docx]

**SUPPLEMENTARY INFORMATION**

“Gene set proximity analysis: expanding gene set enrichment analysis through learned geometric embeddings, with drug-repurposing applications in COVID-19” (Cousins et al.)

Table S1. Description of GEO2KEGG compendium

Table S2. List of semantically similar KEGG gene sets

Table S3. Flow of cohort size with filters

Table S4. Cohort description

Table S1. Description of GEO2KEGG compendium

| **Dataset** | **Disease** | **Disease symbol** |
| --- | --- | --- |
| GSE781 | Renal cancer | KIRC |
| GSE1145 | Dilated cardiomyopathy | DCM |
| GSE1297 | Alzheimer disease | ALZ |
| GSE3467 | Thyroid cancer | THCA |
| GSE3585 | Dilated cardiomyopathy | DCM |
| GSE3678 | Thyroid cancer | THCA |
| GSE4107 | Colorectal cancer | CRC |
| GSE4183 | Colorectal cancer | CRC |
| GSE5281_EC | Alzheimer disease | ALZ |
| GSE5281_HIP | Alzheimer disease | ALZ |
| GSE5281_VCX | Alzheimer disease | ALZ |
| GSE6956AA | Prostate cancer | PRAD |
| GSE6956C | Prostate cancer | PRAD |
| GSE7305 | Endometrial cancer | UCEC |
| GSE8671 | Colorectal cancer | CRC |
| GSE8762 | Huntington disease | HUNT |
| GSE9348 | Colorectal cancer | CRC |
| GSE9476 | Acute myeloid leukemia | LAML |
| GSE11906 | Pulmonary disease chronic obstructive | PDCO |
| GSE14762 | Renal cancer | KIRC |
| GSE14924_CD4 | Acute myeloid leukemia | LAML |
| GSE14924_CD8 | Acute myeloid leukemia | LAML |
| GSE15471 | Pancreatic cancer | PAAD |
| GSE16515 | Pancreatic cancer | PAAD |
| GSE16759 | Alzheimer disease | ALZ |
| GSE18842 | Non small cell lung cancer | LUAD |
| GSE19188 | Non small cell lung cancer | LUAD |
| GSE19420 | Diabetes mellitus type 2 | DMND |
| GSE19728 | Glioma | GBM |
| GSE20153 | Parkinson disease | PARK |
| GSE20164 | Parkinson disease | PARK |
| GSE20291 | Parkinson disease | PARK |
| GSE21354 | Glioma | LGG |
| GSE22780 | Pancreatic neoplasms | PAAD |
| GSE23878 | Colorectal cancer | CRC |
| GSE24739_G0 | Chronic myeloid leukemia | CML |
| GSE24739_G1 | Chronic myeloid leukemia | CML |
| GSE30153 | Lupus erythematosus systemic | LES |
| GSE32676 | Pancreatic cancer | PAAD |
| GSE38666_epithelia | Ovarian neoplasms | OV |
| GSE38666_stroma | Ovarian neoplasms | OV |
| GSE42057 | Pulmonary disease chronic obstructive | PDCO |

Table S2. List of semantically similar KEGG gene sets

| **Gene set 1** | **Gene set 2** |
| --- | --- |
| hsa00100_Steroid_biosynthesis | hsa00140_Steroid_hormone_biosynthesis |
| hsa00510_N-Glycan_biosynthesis | hsa00513_Various_types_of_N-glycan_biosynthesis |
| hsa00532_Glycosaminoglycan_biosynthesis | hsa00533_Glycosaminoglycan_biosynthesis |
| hsa00532_Glycosaminoglycan_biosynthesis | hsa00534_Glycosaminoglycan_biosynthesis |
| hsa00533_Glycosaminoglycan_biosynthesis | hsa00534_Glycosaminoglycan_biosynthesis |
| hsa00601_Glycosphingolipid_biosynthesis | hsa00604_Glycosphingolipid_biosynthesis |
| hsa00601_Glycosphingolipid_biosynthesis | hsa00603_Glycosphingolipid_biosynthesis |
| hsa00603_Glycosphingolipid_biosynthesis | hsa00604_Glycosphingolipid_biosynthesis |
| hsa00982_Drug_metabolism | hsa00983_Drug_metabolism |
| hsa01200_Carbon_metabolism | hsa05230_Central_carbon_metabolism_in_cancer |
| hsa03008_Ribosome_biogenesis_in_eukaryotes | hsa03010_Ribosome |
| hsa04136_Autophagy | hsa04140_Autophagy |
| hsa04210_Apoptosis | hsa04215_Apoptosis |
| hsa04211_Longevity_regulating_pathway | hsa04213_Longevity_regulating_pathway |
| hsa04390_Hippo_signaling_pathway | hsa04392_Hippo_signaling_pathway |
| hsa05204_Chemical_carcinogenesis | hsa05207_Chemical_carcinogenesis |

Table S3. Flow of cohort size with filters

|  | Number of members | Proportion with COVID-19 hospitalization |
| --- | --- | --- |
| All MAPD | 7,727,888 | 13,733 (0.18%) |
| + Enrolled in MAPD during 2019 | 3,757,481 | 10,570 (0.28%) |
| + Received RX claims during 2019-07-01 to 2020-01-31 | 3,353,857 | 10,311 (0.31%) |
| + Enrollment in MAPD during 2019 >= 11 months | 2,978,284 | 8,479 (0.28%) |
| + Enrolled in 2020 | 2,742,888 | 8,448 (0.31%) |
| + Members from NY/NJ/CT | 303,690 | 3,066 (1.01%) |
| **+ Members from the counties identified.** | **234,524** | **2,828 (1**.**21%)** |

Table S4. Cohort description

|  | Cohort (N=234,524) |
| --- | --- |
| **COVID-19 hospitalized** | 2,828 (1.21%) |
| **Age: mean (std)** | 75.13 (9.35) |
| <= 20 | 0 (0.00%) |
| 20–45 | 1,698 (0.72%) |
| 45–65 | 19,853 (8.47%) |
| 65–85 | 181,537 (77.40%) |
| > 85 | 31,436 (13.40%) |
| **Gender** |  |
| Female | 135,497 (57.78%) |
| Male | 99,027 (42.22%) |
| **Race** |  |
| White | 154,704 (65.97%) |
| Black/African-American | 35,096 (14.96%) |
| Asian | 16,587 (7.07%) |
| Other | 14,047 (5.99%) |
| Hispanic/Latinx | 8,483 (3.62%) |
| Unknown | 5,436 (2.32%) |
| Native American/American Indian/Alaska Native | 171 (0.07%) |
| **Comorbidity Indices** |  |
| Hypertension | 170,502 (72.70%) |
| Diabetes without Chronic Complications | 81,040 (34.56%) |
| Anemia Deficiency | 48,625 (20.73%) |
| Diabetes with Chronic Complications | 46,866 (19.98%) |
| Hypothyroidism | 44,515 (18.98%) |
| Peripheral Vascular Disease | 41,789 (17.82%) |
| Chronic Pulmonary Disease | 39,795 (16.97%) |
| Obesity | 30,911 (13.18%) |
| Cerebrovascular Disease | 29,319 (12.50%) |
| Other Neurological Disorders | 26,783 (11.42%) |
| Valvular Disease | 26,660 (11.37%) |
| Solid Tumor without Metastasis | 26,226 (11.18%) |
| Renal Failure | 25,988 (11.08%) |
| Congestive Heart Failure | 24,642 (10.51%) |
| Fluid and Electrolyte Disorders | 19,665 (8.39%) |
| Depression | 16,778 (7.15%) |
| Dementia | 15,181 (6.47%) |
| Liver Disease | 13,563 (5.78%) |
| Rheumatoid Arthritis | 11,035 (4.71%) |
| Weight Loss | 8,597 (3.67%) |
| Psychoses | 6,789 (2.89%) |
| Myocardial Infarction | 6,590 (2.81%) |
| Coagulopathy | 5,797 (2.47%) |
| Paralysis | 4,540 (1.94%) |
| Blood Loss Anemia | 3,828 (1.63%) |
| Metastatic Cancer | 3,079 (1.31%) |
| Pulmonary Circulation Disorder | 2,748 (1.17%) |
| Peptic Ulcer Disease | 2,625 (1.12%) |
| Drug Abuse | 2,457 (1.05%) |
| Alcohol Abuse | 2,450 (1.04%) |
| Lymphoma | 2,202 (0.94%) |
| AIDS/HIV | 973 (0.41%) |
| Moderate to Severe Liver Disease | 701 (0.30%) |
| **Top 5 first 3-digits diagnosis codes (not used in above conditions)** |  |
| e78 (d/o lipoprotein metab lipidemias) | 153,505 (65.45%) |
| m25 (other joint disorder nec) | 54,267 (23.14%) |
| h25 (age-related cataract) | 54,498 (23.24%) |
| m79 (other soft tissue disorders nec) | 53,014 (22.60%) |
| m54 (dorsalgia) | 48,682 (20.76%) |
| **Top 5 AHFS drug therapeutic classes** |  |
| HMG-CoA reductase inhibitors | 116,498 (49.67%) |
| Beta-adrenergic blocking agents* | 71,942 (30.68%) |
| Dihydropyridines | 55,659 (23.73%) |
| Angiotensin II Receptor Antagonists* | 53,098 (22.64%) |
| Angiotensin-Converting Enzyme Inhibitors* | 47,879 (20.42%) |
| **Other features** |  |
| Provider Visit in 2019 | 205,063 (87.44%) |
| Primary Care Provider Visit Count ≥5 in 2019 | 97,313 (41.49%) |
| Primary Care Provider Visit Count ≥11 in 2019 | 22,841 (9.74%) |
| 3 Routine Lab Tests in 2019 | 136,964 (58.40%) |
| IP Stay in 2019 | 27,653 (11.79%) |
| Flu Vaccination in 2019 | 140,760 (60.02%) |
| Count of Unique Drugs ≥6 | 132,974 (56.70%) |
| Count of Unique Drugs ≥10 | 58,215 (24.82%) |
| Count of Unique Drugs ≥14 | 21,962 (9.36%) |
| SNP: Dual Plan | 8,371 (3.57%) |
| SNP: Institutional | 10,221 (4.36%) |
| SNP: Chronic | 391 (0.17%) |

*Summary of covariates:*

1. Age
2. Gender
3. Self-reported race and ethnicity
4. Area-specified SES index based on member zip code
5. 2019 diagnoses as selected from the top 200 first three-digit ICD-10-CM code, excluding codes beginning with “Z”
6. Pre-existing conditions defined by diagnosis codes in 2019, including conditions used in the Charlson Comorbidity Index and Elixhauser Comorbidity Index
7. Pre-existing primary treatment-related diagnosis
8. Co-used prescription drug defined as claims between July 1, 2019, and January 31, 2020, for therapeutic classes
9. Prior hospitalizations in 2019
10. Count of primary care provider visit in 2019
11. Count of unique drugs prescribed
12. Routine screening adherence in 2019, as indicated by completion of a comprehensive metabolic panel, lipid panel, and complete blood count
13. Flu vaccination in 2019 as a proxy of good health behaviors
14. Special Need Plan: (1) institutional, indicating if a member is from a nursing home; (2) dual plan with Medicaid.
